# A Case of Lymphangioma of the Anal Canal

**DOI:** 10.1155/2018/4368131

**Published:** 2018-12-05

**Authors:** Kyriacos Karapashis, Maria Isaia, Antonia Fotiou, Theodora Kalli, Ioannis Michaelides, Nikolaos Nikolaou

**Affiliations:** Surgical Department, Larnaca General Hospital, Cyprus

## Abstract

Lymphangiomas are unusual benign malformations, which can often be misdiagnosed due to their relatively mild and nonspecific symptomatology. Their appearance in the anal canal is extremely rare. Correct diagnosis is necessary for formulating appropriate management. We present the case of a 42-year-old man, with a medical history of ulcerative colitis and mild symptomatology, who was diagnosed with lymphangioma of the anal canal after undergoing a colonoscopy.

## 1. Introduction

Tumors of the anal and perianal region are relatively uncommon, especially mesenchymal benign ones [1]. Lymphangiomas are benign lesions, which can rarely appear in the gastrointestinal tract. Their appearance in the anal canal is extremely rare, with 3 cases being reported so far to our knowledge. Below, we present a case of a 42-year-old man with lymphangioma of the anal canal, treated successfully by surgical excision.

## 2. Case Presentation

**Figure 1:**
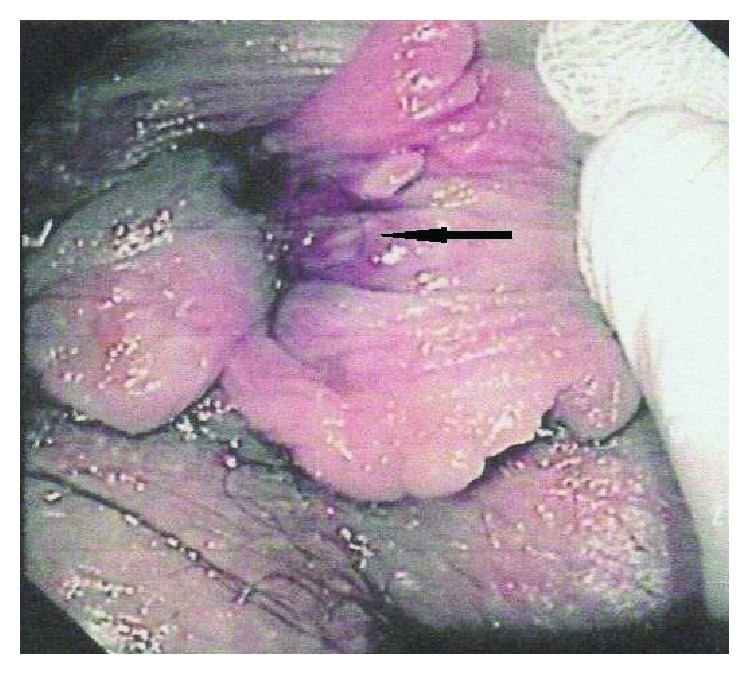
Endoscopic image of the lesion (arrow).

A 42-year-old man with a history of ulcerative colitis was referred to the surgical department by the gastroenterologist, after finding a lesion in the anal canal during follow-up colonoscopy (Figure 1). The patient was monitored by the gastroenterology team for his ulcerative colitis and was being treated with biological factors.

The patient complained of periodic anal pain, discomfort, and rectal bleeding during defecation, which started approximately 6 months earlier. On physical examination, he had an unremarkable abdominal exam. On digital and anoscopic examinations, there were third degree prolapsed hemorrhoids and an ulcerated, soft lesion on top of the 3 o'clock hemorrhoid. Colonoscopy revealed no additional findings from the rest of the rectum and colon. No biopsy was taken during colonoscopy due to the location of the lesion. Consequently, a surgical biopsy of the anal lesion was performed under local anesthesia, as a day case, without any complications. Histopathological examination showed lymphangioma of the anal canal.

**Figure 2:**
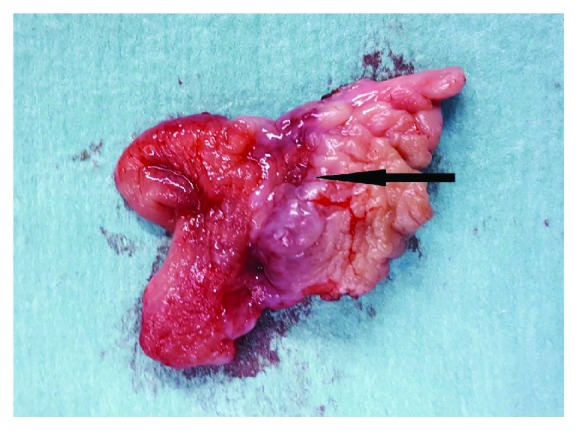
Surgical specimen after wide excision (arrow indicating the ulcerated lesion).

**Figure 3:**
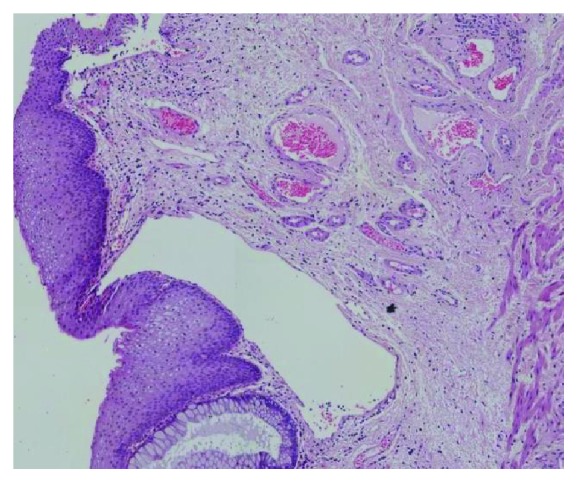
Histopathological image showing dilated lymphatic spaces.

**Figure 4:**
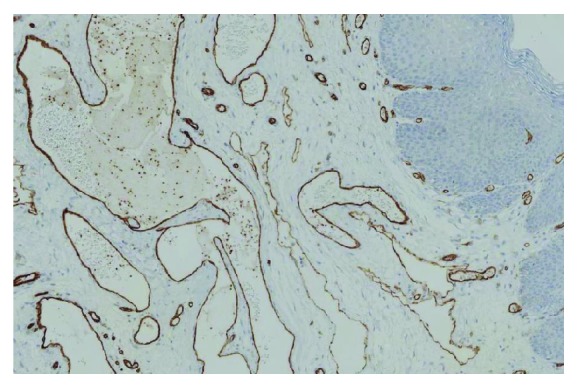
Immunohistochemistry positive for CD-31.

**Figure 5:**
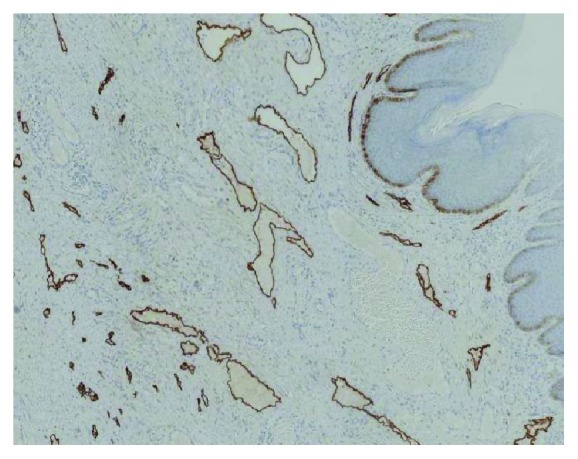
Immune staining positive for D2-40.

A wide excision of the lesion under general anesthesia followed (Figure 2). The new histopathological examination revealed total excision of the lymphangioma, which was a round lesion measuring about 1.5 cm in radius and had at least 0.7 cm of distance from the closest margins of the specimen (Figure 3). Immunohistochemistry was positive for CD31 and D2-40 and negative for CD34 (Figures 4 and 5). At the end of the first postoperative month, full wound healing was accomplished with no signs of recurrence.

## 3. Discussion

Lymphangiomas are uncommon malformations of the lymphatic system that can occur anywhere in the skin and the mucous membranes. They can become evident at any age, but the greatest incidence occurs at birth or early in life. The most common sites are the head and neck, although they can be sometimes found in the intestines, the pancreas, and the mesentery [2].

Cases of lymphangiomas occurring on the lower gastrointestinal tract have been rising in the past years, probably due to the extensive use of specialized diagnostic examinations. So far, lymphangiomas of the anal canal have been extremely rare, since only 3 other cases [1, 3, 4] have been reported to our knowledge. Consequently, little information is available to establish an accepted course of action, in order to properly diagnose and treat potential new cases.

The clinical presentation of lymphangiomas can vary, from asymptomatic to iron deficiency anemia or rectal bleeding [1, 3, 4]. Our patient had vague symptoms of anal discomfort and mild rectal bleeding. He was around 40 years old and had a relatively small in size lymphangioma, data similar to the information reported in the other cases.

Due to the rarity of lymphangiomas, the detection of a tumor in the anal canal always prompts an extensive differential diagnosis. They can be clinically confused with other lesions that can appear in this area such as malignancies and less frequently other benign neoplasms, such as lipomas, papillary hidradenoma, and epidermoid cysts [1]. Also, it is possible to coexist with other lesions, as it was the case with our patient, where it was associated with a nearby hemorrhoid.

The endoscopic findings using magnifying endoscopy with narrow-band imaging techniques could be useful in making the diagnosis of a lymphangioma, as suggested by a report [3]. Mucosal transparency and capillary elongation are identified as unique endoscopic findings that could be a characteristic of a lymphangioma.

As a result, it is suggested by the authors that the diagnosis of lymphangiomas of the anal canal, even though highly unlikely, should be considered when having to differentiate a newfound tumor of the anatomic area. Also, we suggest total excision and biopsy of these tumors as the treatment of choice, as well as any excised lesion of the area, due to the possibility of missing a potential histologic diagnosis. The excision can be done either surgically as in this case or endoscopically as described in other reports, by snare polypectomy [4] or endoscopic submucosal dissection [3]. We await new information about this neoplasm in the years to come that will help the medical community better understand this interesting entity.

## 4. Conclusion

Lymphangioma of the anal canal is a very rare condition but should be considered during differential diagnosis of tumors in the area. In the future, endoscopy could play an important role in the diagnosis of this rare entity. The treatment consists of excision of the lesion, either surgically or by endoscopy.
